# Emerging Role of Extracellular pH in Tumor Microenvironment as a Therapeutic Target for Cancer Immunotherapy

**DOI:** 10.3390/cells13221924

**Published:** 2024-11-20

**Authors:** Md Ataur Rahman, Mahesh Kumar Yadab, Meser M. Ali

**Affiliations:** Department of Oncology, Karmanos Cancer Institute, Wayne State University, Detroit, MI 48201, USA; gm7043@wayne.edu

**Keywords:** tumor microenvironment (TME), extracellular pH, immunotherapy resistance, lactate metabolism, acidic TME, T cells

## Abstract

Identifying definitive biomarkers that predict clinical response and resistance to immunotherapy remains a critical challenge. One emerging factor is extracellular acidosis in the tumor microenvironment (TME), which significantly impairs immune cell function and contributes to immunotherapy failure. However, acidic conditions in the TME disrupt the interaction between cancer and immune cells, driving tumor-infiltrating T cells and NK cells into an inactivated, anergic state. Simultaneously, acidosis promotes the recruitment and activation of immunosuppressive cells, such as myeloid-derived suppressor cells and regulatory T cells (Tregs). Notably, tumor acidity enhances exosome release from Tregs, further amplifying immunosuppression. Tumor acidity thus acts as a “protective shield,” neutralizing anti-tumor immune responses and transforming immune cells into pro-tumor allies. Therefore, targeting lactate metabolism has emerged as a promising strategy to overcome this barrier, with approaches including buffer agents to neutralize acidic pH and inhibitors to block lactate production or transport, thereby restoring immune cell efficacy in the TME. Recent discoveries have identified genes involved in extracellular pH (pHe) regulation, presenting new therapeutic targets. Moreover, ongoing research aims to elucidate the molecular mechanisms driving extracellular acidification and to develop treatments that modulate pH levels to enhance immunotherapy outcomes. Additionally, future clinical studies are crucial to validate the safety and efficacy of pHe-targeted therapies in cancer patients. Thus, this review explores the regulation of pHe in the TME and its potential role in improving cancer immunotherapy.

## 1. Introduction

The TME is a complicated and ever-changing ecosystem that encompasses and interacts with a tumor [1]. The composition of the TME includes a wide variety of cellular and non-cellular constituents, including cancer cells, stromal cells, extracellular matrix (ECM), blood vessels, lymphatic vessels, and signaling molecules [2,3]. TME is a constantly changing and intricate environment that has a crucial impact on the advancement of cancer and the ability to resist therapy [4]. One of the many parameters that contribute to the distinct characteristics of TME is pHe [5]. This parameter has been identified as an important component that influences tumor biology and the effectiveness of treatments [6]. The elevated acidity of the TME, mainly caused by the increased production of lactate, has been linked to several harmful effects on the functioning of immune cells, including the inhibition of anti-tumor immunological responses [7]. The acidity of the TME, also known as acidosis, is caused by the metabolic reprogramming of cancer cells. These cells primarily use glycolysis for energy production, even in the presence of oxygen, which is known as the Warburg effect.

The main cause of TME acidosis is the buildup of lactate, which is a metabolic consequence of glycolysis [8]. Cancer cells undergo glycolysis, converting glucose to lactate, even when there is an adequate supply of oxygen, resulting in the release of lactate into the extracellular environment [9]. Lactate buildup in the TME has two effects: it decreases the pH level (pHe) and influences the immunological composition of the TME [10]. The acidic environment specifically hampers the interaction between activated T cells and tumor cells [11]. Elevated lactate levels can hinder the functioning of T lymphocytes, diminishing their capacity to multiply, generate cytokines, and exert cytotoxic effects on cancerous cells [12]. Furthermore, acidic TME affects the differentiation of macrophages, favoring a transition towards the pro-tumorigenic M2 phenotype [13]. This phenotype facilitates tumor development and inhibits immunological reactions [14].

Given the profound influence of TME acidosis on immune function, targeting lactate metabolism has emerged as a promising strategy in cancer immunotherapy [15]. Strategies to reduce the effects of lactate in the TME entail employing buffer agents to counteract acidity, as well as inhibiting enzymes responsible for lactate generation and transportation [16]. These strategies try to rebalance the pH level, therefore strengthening the effectiveness of immune cells and enhancing therapeutic results [17]. Recent progress in this field has emphasized the possibility of combining pHe regulation with other immunotherapeutic methods, like immune checkpoint inhibitors, to effectively boost anti-tumor immunity in a synergistic manner [18]. Moreover, genes LDHA (Lactate Dehydrogenase A), CA9 (Carbonic Anhydrase 9), MCT4 (Monocarboxylate Transporter 4), GLUT1 (Glucose Transporter 1), HIF-1α (Hypoxia-Inducible Factor 1-alpha), V-ATPase (Vacuolar ATPase), SLC9A1 (Sodium/Hydrogen Exchanger 1, NHE1), ATP6V0C (V-ATPase C Subunit), and PDK1 (Pyruvate Dehydrogenase Kinase 1) have been linked to the control of extracellular acid generation and adjustment inside the TME [19]. These genes are involved in various metabolic pathways, including glycolysis, lactate transport, and the regulation of pH [20].

Nevertheless, the therapeutic adjustment of TME pH has notable difficulties, such as the possibility of unintended effects and the intricacy of precisely targeting the TME while avoiding harm to healthy tissues [21]. Successfully addressing these obstacles will be essential for the seamless incorporation of pHe modulation techniques into clinical practice [22]. To summarize, the increasing importance of pHe in the tumor TME as a target for cancer immunotherapy is a rapidly growing area with considerable promise [23]. Researchers seek to optimize the effectiveness of immune-based medicines and improve patient outcomes by comprehending and controlling the acidic microenvironment of tumors [24,25]. This review emphasizes further investigation in this area that shows potential for the advancement of groundbreaking cancer therapies that utilize the distinct characteristics of the TME targeting cancer immunotherapies.

## 2. Extracellular pH Production in the Tumor Microenvironment

The Warburg effect refers to the phenomena where cancer cells display abnormal metabolism, characterized by elevated glycolysis and lactate generation, even when oxygen is available [26]. The metabolic transition leads to the accumulation of lactate and protons outside of cells, causing a decrease in pHe [27]. The acidification becomes more severe by the hypoxic circumstances often present in tumors, which stimulate the production of carbonic anhydrase IX (CAIX) and other proton transporters that help remove acid [28].

### 2.1. Lactate Production in the Tumor Microenvironment (Acidosis)

**Figure 1:**
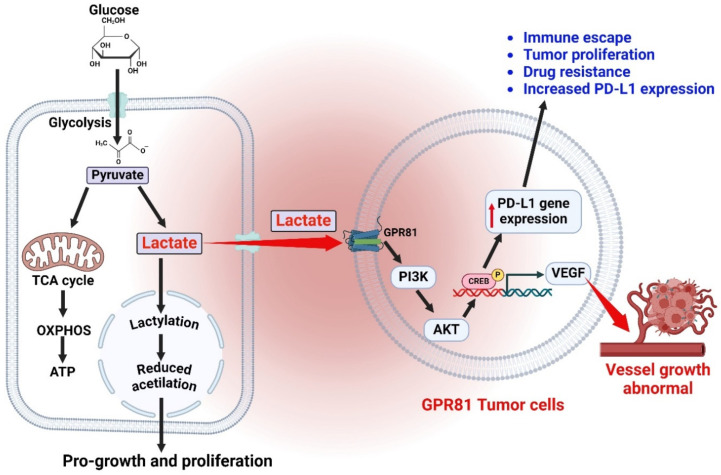
Lactate production in proliferating and tumor cells expressing GPR81. The metabolic process in rapidly dividing tumor cells that express GPR81 results in the generation of lactate and consequent acidosis in the surrounding tumor microenvironment. Tumor cells, because of their rapid pace of growth, primarily depend on glycolysis for generating energy, even when oxygen is available (known as the Warburg effect). This metabolic transition leads to the conversion of glucose into pyruvate, which is then converted into lactate by the action of lactate dehydrogenase (LDH). The surplus lactate generated is expelled from the cell via monocarboxylate transporters (MCTs), resulting in acidic extracellular surroundings. The figure was created using BioRender.com.

Lactate has a crucial role in the TME, acting as both a source of energy and a chemical that can affect several cellular processes through signaling [29]. High levels of lactate in the context of cancer are linked to a negative outlook and reduced responsiveness to treatment [30]. Lactate could regulate the function of immune cells, such as T cells and macrophages, which in turn promotes an immunosuppressive TME [31]. Glucose uptake is enhanced through the utilization of glucose transporters (GLUTs) [32]. Glucose undergoes metabolic processes in the cytoplasm to be transformed into pyruvate, resulting in the production of ATP and NADH [33]. LDH catalyzes the conversion of pyruvate to lactate. Mono-carboxylate transporters (MCTs) facilitate the export of lactate from the cell, leading to a buildup of lactate in the TME [34]. The buildup of these substances results in a reduction in the pH of the surrounding environment, leading to the creation of an acidic microenvironment [35]. The GPR81 receptor, which is a large family of membrane proteins involved in transmitting signals from the extracellular environment into the cancer cell, is stimulated by elevated levels of lactate in the surrounding environment [34]. Activation of GPR81 can intensify glycolytic activity and promote the generation of lactate, which sustains acidic conditions [36]. The acidic microenvironment has a significant impact on different aspects of tumor biology [37]. It promotes cell survival, invasion, angiogenesis, immunological suppression, and drug resistance [38,39]. As a result, it contributes to the growth of tumors and their resistance to therapy [40] (Figure 1).

### 2.2. Interaction Between Activated T Cells and Tumor Cells

**Figure 2:**
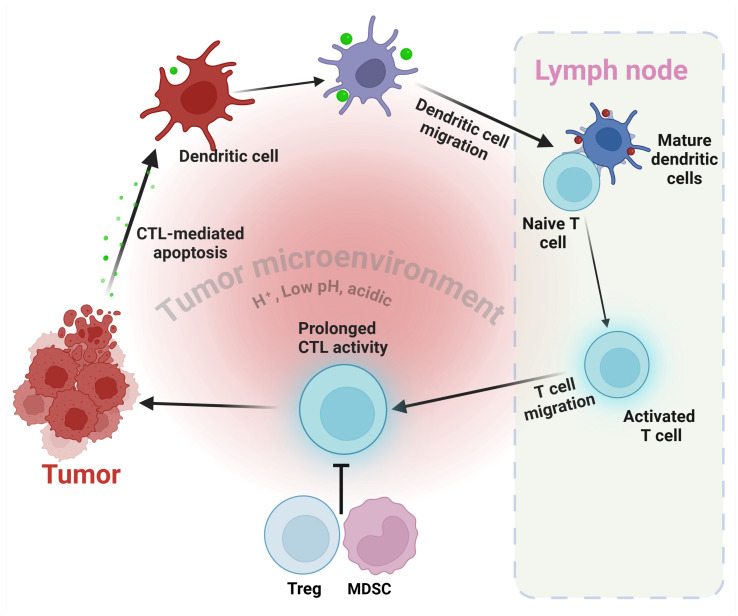
Tumor-specific T cell induction and activation. This diagram depicts the fundamental mechanisms involved in the activation and operation of tumor-specific T cells within the TME. Antigen presentation by dendritic cells (DCs), which relocate from the tumor to the lymph nodes. In this context, DCs stimulate the activation of T cells, namely cytotoxic T lymphocytes (CTLs), by presenting antigens derived from tumors. Inducing CTL activity results in the apoptosis of tumor cells, which is carried out by CTLs. Suppressive functions of myeloid-derived suppressor cells (MDSCs) and regulatory T cells (Tregs) in regulating immunological responses. The intricate interaction within the TME ultimately affects the effectiveness of the immune response against the tumor. The figure was drawn and modified by BioRender.com, 6 November 2024.

The acidic TME presents a substantial obstacle to the efficacy of T cells, which play a vital role in the immune response against tumors [41]. Acidic conditions can hinder the functioning of T cell receptor (TCR) signaling, cytokine synthesis, and cytotoxic action [42]. In addition, the buildup of lactate can impede the differentiation and activity of cytotoxic T lymphocytes (CTLs), hence enhancing the ability of malignancies to evade the immune system [43] (Figure 2).

**Figure 3:**
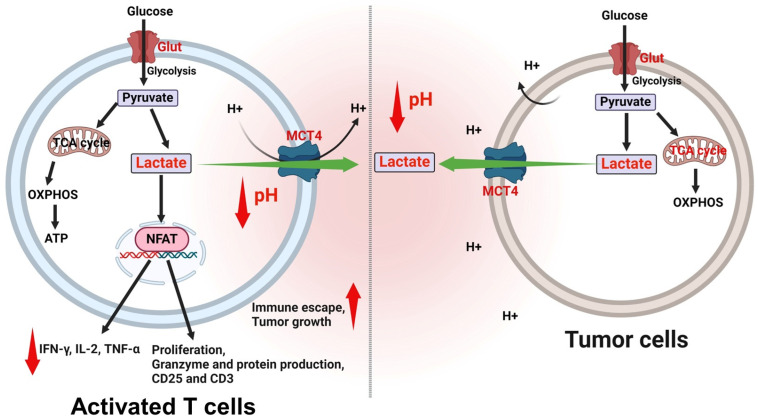
Effect of low pH on activated T cell and tumor cell interactions. Elevated lactate levels from tumor cells hinder T cell activation, proliferative capacity, and cytotoxic effect, diminishing their capacity to specifically target and eliminate tumor cells. Acidic environments inhibit T cell activity, which suppresses T cells’ anti-tumor response and lets tumor cells avoid immune monitoring and multiply. This indicates that acidosis promotes tumor survival, proliferation, and resistance to immunotherapy. The figure was created using BioRender.com.

Lactate exerts a multifaceted influence on the behavior of T cells within the TME [44]. Elevated lactate levels can disrupt the redox equilibrium in T cells, resulting in reductive stress that hampers their functionality and survival [45]. Increased levels of lactate can hinder the multiplication of T cells, leading to a decrease in the total number of T cells that can initiate a strong immunological response [46]. Lactate could inhibit the synthesis of important cytokines, including IL-2 and IFN-γ, which play a critical role in the activation and functioning of T cells [47]. Lactate could selectively hinder the cytotoxic activities of CD8^+^ T lymphocytes, which are crucial in identifying and eliminating cancer cells [48]. The presence of an acidic environment, primarily caused by the buildup of lactate, can additionally hinder the functioning of T cells and facilitate the evasion of the immune system by tumors. Lactate could decrease the function of NFAT, which is a transcription factor that plays a crucial role in the activation of T cells and the production of cytokines [49] (Figure 3). High lactate levels impair the activation and function of both T cells and natural killer (NK) cells, hence compromising the immune response against malignancies [50]. Treatment with lactate can increase the expression of genes related to T cell function and signaling, perhaps augmenting some elements of T cell activity [51]. Lactate could stimulate HCAR1 (sometimes referred to as GPR81), a receptor that could potentially be utilized in specific tumor treatments [52]. Activation of HCAR1 has been linked to a range of metabolic and immune-regulatory mechanisms that can be advantageous in certain situations [53]. Developing a comprehensive understanding of the dual function of lactate in the TME is essential for the development of therapeutic approaches that can either reduce its detrimental impacts or utilize its beneficial effects to enhance the body’s immune response against tumors [54].

### 2.3. Effect of Lactic Acid on Macrophage Polarization

Macrophages are a highly adaptable part of the immune system that can change their functional characteristics in response to environmental signals [55], thus recognizing, engulfing, and eliminating infections, cellular debris, and cancer cells [8]. They derive from monocytes, a category of white blood cells, and undergo differentiation upon tissue entry [56]. Macrophages exhibit plasticity, assuming either pro-inflammatory (M1) or anti-inflammatory (M2) phenotypes in response to stimuli from the environment [57]. Essential functions encompass phagocytosis, antigen presentation, and cytokine release, which are vital to adaptive as well as innate immunity. Macrophages are categorized as tissue-resident macrophages, such as alveolar macrophages in the lungs and Kupffer cells in the liver, and inflammatory macrophages that are recruited during infections [58]. They facilitate wound healing, immunological control, and the maintenance of homeostasis.

**Figure 4:**
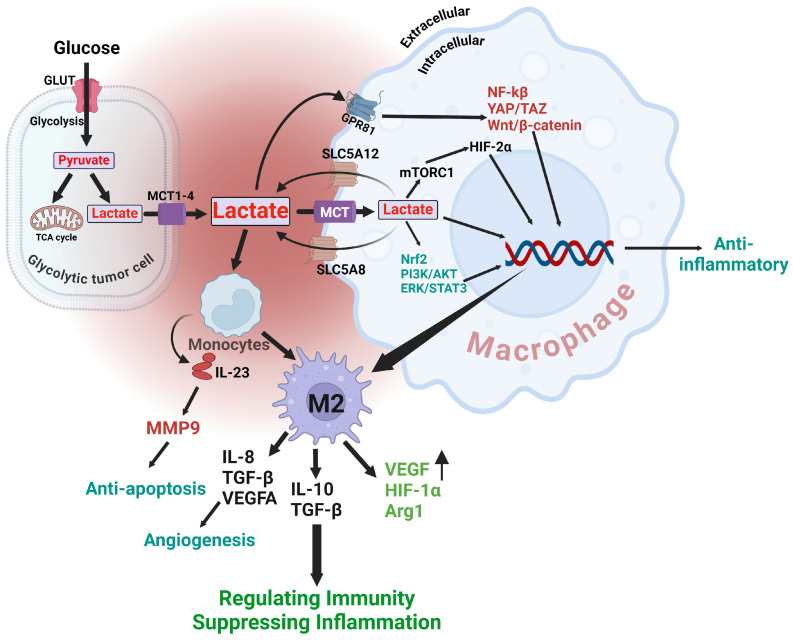
The role of lactic acid in macrophages during inflammation as a signaling molecule. Lactic acid communicates with macrophages by two main mechanisms: transporter-mediated (McT) or sodium-dependent co-transport (SLC5A8, SLC5A12), and receptor-mediated (GPR). Lactic acid employs a negative feedback mechanism to impede glycolysis. Lactic acid stimulates the Wnt/β-catenin and Yap/Taz signaling pathways and suppresses the NF-κB signaling pathway via binding to GPR receptors on the cell membrane. Lactic acid penetrates the nucleus and attaches to histone lysine residues, so directly stimulates the activation of M2 genes by histone lactylation. The figure was created using BioRender.com.

Studies have demonstrated that lactate in the TME might cause macrophages to adopt an M2-like phenotype, which is associated with immunosuppression [59]. Tumor-associated macrophages (TAMs) facilitate tumor progression by stimulating the formation of new blood vessels (angiogenesis), modifying tissue structure, and inhibiting the body’s adaptive immune responses [60]. Lactic acid, a substance produced during the breakdown of glucose without oxygen, has a notable impact on the differentiation of macrophages in the specific environment of a tumor [61]. A macrophage is a type of white blood cell that plays a crucial role in the immune system by engulfing and destroying foreign substances, such as bacteria and polarization can manifest as two distinct phenotypes [62]. M1 Macrophages, which are pro-inflammatory, play a role in anti-tumor responses by generating pro-inflammatory cytokines and reactive oxygen species (ROS) that eliminate tumor cells [63]. M2 Macrophages, which have anti-inflammatory properties, contribute to tissue repair and tumor growth by releasing anti-inflammatory cytokines, facilitating angiogenesis, and modifying the extracellular matrix [64]. Lactic acid induces the polarization of macrophages towards the M2 phenotype [61]. This shift is advantageous for the proliferation and survival of tumors, as M2 macrophages facilitate immune suppression, angiogenesis, and tissue remodeling [60]. Macrophages undergo metabolic alterations in response to lactic acid. Elevated concentrations of lactic acid result in heightened glycolysis and modified oxidative phosphorylation, promoting the M2 phenotype [65]. Lactic acid acts as a stabilizer for hypoxia-inducible factor 1-alpha (HIF-1α), which is a transcription factor responsible for activating genes related to the M2 phenotype [66]. Lactic acid enhances the synthesis of anti-inflammatory cytokines, namely IL-10 and TGF-β, which serve as indicators of M2 macrophages [67]. Additionally, it reduces the synthesis of pro-inflammatory cytokines such as IL-12 and TNF-α. Lactic acid plays a crucial role in promoting M2 macrophage polarization, contributing to immunosuppressive TME (Figure 4). This allows tumor cells to avoid being detected and destroyed by the immune system. Comprehending the function of lactic acid in macrophage polarization can offer valuable knowledge for creating therapeutic approaches that focus on metabolic pathways to regulate the immune response in the TME [68]. This has potential to improve the efficacy of immunotherapies by tilting the equilibrium towards a stronger pro-inflammatory and anti-tumor immune response.

## 3. Targeting Lactate Metabolism for Cancer Immunotherapy

Cancer immunotherapy is a treatment approach that boosts the immune system’s capacity to recognize and eliminate cancer cells [69]. Given lactate’s impact on the immunosuppressive TME, targeting lactate metabolism offers promising potential to enhance the effectiveness of cancer immunotherapy [70]. Researchers are currently exploring inhibitors of LDH, monocarboxylate transporters (MCTs), and other key enzymes involved in lactate production and transport [71]. These techniques target the reduction in lactate levels in the TME with the goal of enhancing immune cell function and increasing the effectiveness of immunotherapies. Table 1 presents the most recently used medications that specifically target lactate metabolism for the purpose of cancer immunotherapy within the TME.

## 4. Recent Targeting Extracellular pH in Cancer Immunotherapy

In addition to lactate metabolism, the direct manipulation of pHe offers another potential approach for therapy [81]. Preclinical research has demonstrated the promise of approaches such as buffering the TME, blocking proton pumps, or targeting CAIX [82]. These treatments try to regulate the pH level, therefore reducing the negative impact of acidity on immune cells and improving the immune response against tumors [83]. The pHe, often known as the acidity or alkalinity of the environment around cells, has a notable impact on the advancement of cancer and the effectiveness of immunotherapy [84]. Tumor cells exhibit a higher level of acidity in comparison to healthy cells [83]. Tumors generate an acidic microenvironment that impairs the activity of immune cells, reducing their ability to recognize and kill cancer cells [85]. Additionally, this environment facilitates the growth and spread of tumors [86]. Therefore, focusing on the pHe of tumors is a developing approach to enhance the efficacy of cancer immunotherapy [87]. Currently, researchers have been employing various strategies to specifically target pHe in cancer immunotherapy [88]. Table 2 shows medications altering the acidic TME to improve cancer immunotherapies. However, doses and applications vary by therapeutic context and often incorporate combination therapy [89].

### 4.1. Utilizing Buffer Agents

**Figure 5:**
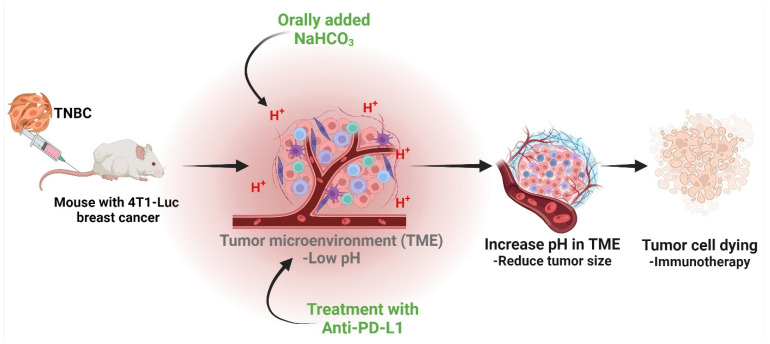
Effect of targeting extracellular tumor pH on immunotherapy efficacy in a triple-negative breast cancer (TNBC) mouse model. Breast cancer cells release lactate and protons to maintain intracellular homeostasis, resulting in an acidic pHe in the tumor microenvironment. Oral administration of sodium bicarbonate or sodium bicarbonate plus anti-PD-L1 combination enhances anti-tumor immunity in TNBC. Treatment leads to tumor growth inhibition and increased survival time, indicating the potential of pH-neutralizers in improving cancer immunotherapy. This combination therapy demonstrates significant impact, offering a powerful approach to personalized medicine. The figure was created using BioRender.com.

Buffering agents could counteract the acidic tumor environment, hence creating a more conducive setting for immune cells to operate effectively [105]. Our recent study found that administering sodium bicarbonate (NaHCO_3_) orally improved the effectiveness of anti-PD-L1 antibody treatment in combating tumors [92]. This was demonstrated by the observed induction of anti-tumor immunity, suppression of tumor development, and enhancement of survival in the 4T1-Luc breast cancer model. Our observation revealed that NaHCO_3_ caused a rise in the pHe in tumor tissues in living organisms. This increase in pH was associated with an augmentation in the infiltration and activation of T cells, as well as an elevation in the mRNA expression of IFN-γ, IL-2, and IL-12p40 in tumor tissues. Furthermore, there was an augmentation in the activation of T cells in the lymph nodes that drain the tumor. Significantly, these effects were enhanced when NaHCO_3_ was paired with anti-PD-L1 therapy. Under this condition, the presence of acidity in the external environment greatly enhanced the expression of PD-L1. The results indicate that using NaHCO_3_ as a therapy to increase alkalinity shows promise as a new way to modulate the immune response in the TME (Figure 5). Our hypothesis is that the administration of NaHCO_3_ can enhance the anti-cancer effects of anti-PD-L1 therapy specifically in breast cancer. This combined treatment has the potential to greatly influence future immunotherapeutic methods for triple-negative breast cancer (TNBC) by offering a strong customized medicine approach. The findings of our study have significant implications for enhancing the outcomes of patients with TNBC.

### 4.2. Enzymatic Inhibition

In cancer immunotherapy, an exciting approach involves the inhibition of enzymes to target the pHe [106]. TME frequently displays acidic pH levels resulting from aberrant metabolic activities, such as heightened glycolysis and lactate synthesis [107]. The presence of an acidic environment might hinder the proper functioning of immune cells, hence facilitating immune evasion and making the body less responsive to treatment [108]. By suppressing the activity of enzymes involved in the generation of acid, such as CAIX and lactate dehydrogenase (LDH), it is feasible to restore the normal pH level, thus improving the efficacy of immune responses against malignancies [109]. Enzyme inhibition can decrease the acidity of the tumor microenvironment, potentially enhancing the infiltration and functionality of immune cells, such as T cells and natural killer cells [110]. Moreover, maintaining a balanced pHe can improve the effectiveness of immune checkpoint inhibitors and other immunotherapies, as they tend to be less potent in acidic environments [111]. The approach of targeting pHe by inhibiting enzymes provides a twofold advantage: it directly hampers the growth of malignancies by affecting cellular metabolism and enhances the vulnerability of cancers to immunological assault [105]. This versatile strategy has considerable potential for addressing the obstacles of immune resistance in cancer treatment, creating a more conducive environment for successful immunotherapy [112]. Table 3 outlines the functions of different enzymes in controlling the acidic environment of tumors and their potential as targets to improve the effectiveness of cancer immunotherapy.

## 5. Genes Involved in Extracellular TME Acid Production and Modulation

TME becomes acidic due to changes in the metabolism of cancer cells, resulting in the generation and buildup of acidic metabolites [83]. Multiple genes and enzymes participate in this process, leading to the acidity in the TME [121]. Some important genes and their corresponding enzymes are discussed. LDHA facilitates the transformation of pyruvate into lactate, which significantly contributes to the increase in extracellular acidity, especially under low oxygen situations [122]. Subsequently, lactate is extruded from the cell, so adding to the acidic milieu [123]. Transporters MCT1 and MCT4, also known as Monocarboxylate Transporters 1 and 4, respectively [124], facilitate the removal of lactate and protons from the cells [125]. MCT1 and MCT4 are essential for maintaining the acidic environment in cancer cells by facilitating the export of lactate, which is a byproduct of glycolysis [126]. CA9 and CA12 enzymes, also known as Carbonic Anhydrases IX and XII, respectively, facilitate the reversible process of converting carbon dioxide into bicarbonate and protons through hydration [127]. CA9 and CA12 are frequently increased in hypoxic environments, aiding in the preservation of an acidic pHe by enhancing the release of protons [113]. GLUT1, also known as Glucose Transporter 1, is a protein that facilitates the transport of glucose molecules [128]. GLUT1 indirectly enhances glucose absorption into cells, supporting glycolysis and subsequent lactate synthesis, while it does not directly participate in acid generation [129]. The Warburg effect, which is defined by heightened glycolysis even in the presence of sufficient oxygen, plays a role in the generation of acid [130]. V-ATPase, also known as Vacuolar ATPase, is an enzyme [131]. This proton pump has a role in moving protons across the membranes inside cells and on the surface, which leads to the acidification of compartments within cells and the external environment [132]. The genes and their corresponding proteins play a vital role in the metabolic reprogramming of cancer cells [133], resulting in acidic TME (Table 4). This acidic TME can have an impact on the advancement of cancer, its spread to other parts of the body, and how it responds to treatment [134].

## 6. Targeting Extracellular pH in Clinical Trials

Targeting extracellular pH has emerged as a promising treatment method, with numerous approaches currently under investigation in clinical studies. One method includes buffering agents like sodium bicarbonate. Sodium bicarbonate has demonstrated the ability to neutralize tumor acidity and is currently undergoing evaluation in Phase I/II clinical trials (NCT03420480) for multiple cancer forms, including pancreatic cancer [146]. Preclinical studies suggest that alkalinizing the tumor microenvironment may diminish metastasis and enhance the effectiveness of chemotherapeutics by inhibiting drug breakdown in acidic circumstances [147]. A separate category of pH-targeting pharmaceuticals comprises inhibitors of carbonic anhydrase IX (CAIX), an enzyme that is overexpressed in tumors to facilitate pH regulation and improve survival in hypoxic environments [148]. Agents such as SLC-0111 have progressed to clinical trials (NCT02215850) to evaluate their efficacy in inhibiting CAIX activity and enhancing tumor sensitivity to treatment [149]. Preliminary findings indicate that inhibiting CAIX may suppress tumor proliferation and improve responses to conventional cancer therapies. Proton pump inhibitors, including omeprazole and lansoprazole, are being repurposed for oncological treatment to inhibit proton pumps and elevate pHe [150]. They have been assessed in multiple clinical trials, including NCT01198821, aimed at improving the efficacy of chemotherapeutic drugs in breast and gastric malignancies. The VATPase inhibitor esomeprazole is approved for clinical use and has been used in cancer studies [151]. The MCT1 inhibitor AZD3965 is undergoing clinical trials (NCT01791595) [152]. A possible technique entails the use of V-ATPase inhibitors (e.g., esomeprazole) that interfere with proton gradients across membranes, so directly affecting intracellular and pHe [153]. These inhibitors are being studied for their capacity to sensitize cancer cells to apoptosis, with current trials examining their therapeutic efficacy. Finally, pH-responsive nanoparticle systems are being engineered to selectively release medications in the acidic TME [154]. Clinical trials are currently assessing nanomedicines to enhance drug delivery and minimize off-target effects. In summary, the modulation of pHe by buffering agents, CAIX inhibitors, proton pump inhibitors, and pH-responsive drug delivery systems is now under investigation in clinical studies. These strategies show potential to surmount resistance mechanisms and improve the effectiveness of current cancer treatments.

## 7. Focusing Extracellular pH in Existing Cancer Treatment

The extracellular pH and its influence on immunotherapy resistance in breast cancer, along with the incorporation of pHe-targeting techniques into existing cancer therapies, is essential. Consider the following essential issues about time and treatment synergies: Decreasing pHe before chemotherapy or immunotherapy may enhance the sensitivity of cancer cells, hence augmenting medication absorption and immune response. Bicarbonates or PPIs may be provided prior to treatment to neutralize acidic pH, potentially augmenting the efficacy of immunotherapies such as checkpoint inhibitors [92]. pHe-targeting medicines can be co-administered with conventional therapies, including chemotherapy. Integrating pH-modulating treatments with doxorubicin, paclitaxel, or cisplatin may enhance cytotoxicity [155]. Acidic microenvironments enhance radiation resistance; pHe neutralization can increase the radiosensitivity of cancer cells [156]. Coordinating the administration of pH modulators with these therapies guarantees prolonged synergy during maximum medication efficacy. Administering pH buffers after therapy may avert relapse by obstructing the acidic milieu that promotes cancer stem cell viability and tumor recurrence [157]. Ongoing pH manipulation may enhance immunological memory, hence aiding in the prevention of metastasis or subsequent cancers. Personalized tumor profiling for pHe dynamics may enhance the timing and combination of therapeutic interventions. Modifying the order of immunotherapy and pHe modification may yield improved therapeutic results. Administering pH modulators promptly following immune checkpoint blockage may inhibit the re-establishment of an immunosuppressive acidic microenvironment [158]. These methodologies offer a framework for formulating pHe-targeting combination medicines and integrating them with traditional methods for enhanced cancer therapy efficacy.

## 8. Future Directions and Clinical Applications of Extracellular pH in Cancer Immunotherapy

The potential of cancer immunotherapy targeting pHe normalization to boost treatment efficacy and overcome resistance makes it a viable future direction [159]. Present studies concentrate on the creation of inhibitors for enzymes including CAIX, LDH, and monocarboxylate transporters (MCTs), which play a crucial role in sustaining the acidic TME [160]. These inhibitors are designed to regulate the pH level, thereby producing an optimal environment for immune cells to operate efficiently [161]. One potential use of this method is to combine enzyme inhibitors with current immunotherapies, such as immune checkpoint inhibitors and adoptive cell treatments, in clinical settings [162]. Optimizing the pH level can enhance the infiltration and activity of immune cells, increase the effectiveness of treatments such as anti-PD-1 and anti-CTLA-4 antibodies, and potentially decrease immune-related side effects by stabilizing the TME [163]. Moreover, this approach may be advantageous in solid tumors characterized by elevated metabolic activity and acidity, which frequently exhibit resistance to standard therapies [164]. Subsequent clinical trials are expected to investigate the most effective dosage, combination approaches, and criteria for selecting patients to maximize the therapeutic advantages of pHe normalization in cancer immunotherapy [165]. This strategy signifies a noteworthy advancement in individualized cancer therapy, with the capacity to enhance patient results and expand the usefulness of immunotherapies [166].

## 9. Limitations in Therapeutic Perspective in Cancer Immunotherapy

Although there are promised benefits in targeting pHe normalization in cancer immunotherapy, it is necessary to resolve numerous challenges. A major challenge is the diversity of tumors, as different types of cancers and even different parts within the same tumor might exhibit varied degrees of acidity (pHe) and metabolic characteristics. The heterogeneity in enzyme inhibitors, such as CAIX or LDH, can impact their effectiveness, making it challenging to universally anticipate therapy outcomes [167]. In addition, the systemic administration of these inhibitors may result in unintended consequences and toxicity in normal tissues, which also depend on comparable enzymes for their regular functions. For instance, the inhibition of CAIX could disturb the typical acid–base equilibrium in healthy organs, resulting in potential adverse effects [168]. One further constraint is the cancer cells’ ability to adapt, perhaps leading to resistance against pHe-modulating treatments in the long run. This flexibility has the potential to diminish the long-term efficacy of such therapies. Moreover, the presence of an acidic microenvironment not only impacts the functioning of immune cells but also influences various other aspects of tumor biology, including drug resistance and metastasis [169], thereby making treatment methods more complex. Finally, the incorporation of pHe normalization into current treatments necessitates meticulous evaluation of dosage and timing to prevent antagonistic interactions. These constraints emphasize the necessity in conducting thorough clinical research to improve therapeutic methods and create biomarkers for categorizing patients.

## 10. Conclusions

The TME is essential in the advancement of cancer and the effectiveness of treatment, with pHe being a critical determinant. The acidification of the tumor microenvironment, mainly caused by the end point of glycolysis lactate, might hinder the functioning of immune cells, specifically by inhibiting the activation of T cells and encouraging the polarization of immunosuppressive macrophages [59,170]. The presence of this antagonistic milieu reduces the effectiveness of the immune system’s response to cancerous cells. Targeting lactate metabolism has become a promising approach in cancer immunotherapy, with the goal of restoring immune activity by normalizing the pH levels. Preclinical investigations have demonstrated the promise of approaches such as employing buffer agents and blocking enzymes involved in lactate generation. Moreover, comprehending the genetic control of extracellular acid production could present novel therapeutic objectives [171]. Nevertheless, the implementation of clinical applications encounters difficulties, such as the intricacy of the TME and the possibility of adverse reactions. Subsequent investigations should prioritize the improvement of these strategies, the enhancement of their compatibility with current medicines, and the identification of biomarkers to classify patients. The incorporation of pHe regulation into cancer immunotherapy is a new and interesting approach, but it needs additional validation and clinical research to properly understand its therapeutic effectiveness. As research advances in this field, it is expected that focusing on the pH outside of cells will become a crucial part of future cancer immunotherapies.

## Figures and Tables

**Table 1:** TME lactate metabolism medicines for cancer immunotherapy.

Drug Name	Dose	Mode of Action	Application	References
Dichloroacetate (DCA)	10–50 mg/kg/day	Inhibits pyruvate dehydrogenase kinase, shifts metabolism to oxidative phosphorylation	Used to reduce lactate production in cancer cells	[72]
Metformin	500–2550 mg/day	Inhibits mitochondrial complex I, reduces lactate production	Anti-diabetic drug, repurposed for cancer treatment	[73]
2-Deoxyglucose (2-DG)	25–50 mg/kg/week	Inhibits glycolysis, leading to reduced lactate production	Used to sensitize tumors to radiotherapy and chemotherapy	[74]
AZD3965	5–10 mg/kg/day	Inhibits monocarboxylate transporter 1 (MCT1), reduces lactate export	Under investigation in solid tumors	[75]
LDHA Inhibitor (FX11)	50 mg/kg/day	Inhibits LDHA, reducing lactate production	Used to induce metabolic stress in cancer cells	[76]
Galloflavin	10–20 mg/kg/day	Inhibits lactate dehydrogenase, reducing lactate production	Investigational agent in various cancers	[77]
IACS-10759	100 mg/day	Inhibits oxidative phosphorylation, leading to metabolic stress	Under investigation in AML and solid tumors	[78]
Oxamate	1–2 g/kg/day	Inhibits lactate dehydrogenase, reducing lactate production	Investigational agent in combination with other therapies	[79]
3-Bromopyruvate (3-BP)	50–100 mg/kg/day	Alkylates thiol groups in glycolytic enzymes, reducing lactate production	Experimental agent in glycolysis inhibition	[80]
Gossypol	10–70 mg/day	Inhibits LDHA and Bcl-2 family proteins, reducing lactate production	Used in combination therapy for various cancers	[80]

**Table 2:** Recent drug targets to modulate pHe in the TME for cancer immunotherapy.

Drug Name	Dose	Molecular Mechanism	Application	References
Acetazolamide	250–500 mg/day	Reduces bicarbonate and acid output by inhibiting carbonic anhydrase	Reducing tumor acidity, enhancing immune cell function	[90,91]
Sodium Bicarbonate	0.5–1 g/kg (oral)	Buffers extracellular pH, neutralizing acidity	Alleviating acidic TME, improving drug efficacy	[92]
Omeprazole	20–40 mg/day	Proton pump inhibitors lower gastric acid secretion and tumor acidity.	Adjunct in cancer therapy, modulating TME acidity	[93,94]
Esomeprazole	20–40 mg/day	Proton pump inhibitor, reduces extracellular acidification	Combination with immunotherapy, enhancing T cell function	[93]
Bafilomycin A1	Variable (research)	By inhibiting V-ATPase, proton extrusion and extracellular acidification are reduced	Experimental, targeting acidic microenvironment	[95,96]
AZD7986	3–300 mg/kg (clinical trials)	Inhibits CAIX, reducing extracellular acidity	Targeting hypoxic tumor regions, improving immune response	[97,98]
Sodium Dichloroacetate (DCA)	10–50 mg/kg	Pyruvate dehydrogenase kinase inhibition alters metabolism and reduces lactate generation	Modulating tumor metabolism, reducing acidosis	[72,99]
5-Aminolevulinic Acid (ALA)	2–20 mg/kg	Produces heme; targets acidic cancers with photodynamic treatment	Combined with light exposure for targeted tumor ablation	[100,101]
Bromopyruvate	Variable (research)	Reduces tumor acidity and lactic acid generation by inhibiting glycolytic enzymes.	Experimental, targeting glycolytic tumors	[72,102]
CPI-613	400–3000 mg/m^2^ (IV)	Alters tumor metabolism by inhibiting energy-producing mitochondrial enzymes	In combination with chemotherapy for enhanced effect	[103,104]

**Table 3:** Essential enzymes, their molecular actions, roles, and therapeutic applications for cancer pHe homeostasis.

Enzyme	Molecular Action	Function in pHe Regulation	Immunotherapy Application	References
Carbonic Anhydrase IX (CAIX)	Facilitates the reversible conversion of CO_2_ into bicarbonate (HCO_3_⁻) and protons (H⁺) through a chemical reaction	Contributes to the process of extracellular acidification by actively transporting H⁺ ions out of the cell	Tumor acidity is decreased by inhibition, which improves the functioning of immune cells and their response to checkpoint inhibitors	[113,114,115]
Lactate Dehydrogenase (LDH)	Converts pyruvate to lactate, producing NAD⁺	Increases lactate production, leading to extracellular acidification	Inhibitors of LDH can reduce lactate levels, leading to the normalization of pH levels and the enhancement of the immunological microenvironment	[116,117]
Monocarboxylate Transporter (MCT)	Facilitate the movement of lactate and protons across cellular membranes	Lactate and H⁺ exports contribute to extracellular acidification	Lactate exports are reduced by MCT inhibitors, altering acidity and immunological response	[118,119]
V-ATPase (Vacuolar-type H⁺-ATPase)	Pumps protons into the extracellular space or into intracellular vesicles	Major controller of internal and pHe levels in tumors	Suppression of acidity enhances the ability of immune cells to enter and function effectively	[120]

**Table 4:** Extracellular TME acid-producing genes and their molecular processes and functions.

Gene	Molecular Mechanism	Function	References
LDHA	Catalyzes the conversion of pyruvate to lactate	Increases extracellular acidity, especially under hypoxic conditions by producing lactate	[135,136]
MCT1	Facilitates the export of lactate and protons from cells	Maintains acidic environment by removing lactate, a glycolysis byproduct	[137,138]
MCT4	Same as MCT1, facilitates lactate and proton export	Supports the acidic TME by removing lactate from cancer cells	[119,138]
CA9	Converts CO_2_ to bicarbonate and protons	Enhances proton release, contributing to extracellular acidity, particularly in hypoxic conditions	[139,140]
CA12	Similar function to CA9	Helps maintain acidic pHe through proton release	[140,141]
GLUT1	Transports glucose into cells	Supports glycolysis and subsequent lactate production, indirectly contributing to TME acidity	[142,143]
V-ATPase	Transports protons across cellular and vesicular membranes	Acidifies intracellular compartments and the external environment, contributing to extracellular acidity	[83,144,145]

## Data Availability

All necessary data generated or analyzed during this study are presented in this article and additional data could be available from the corresponding author upon request.
